# Trends in Food Preferences and Sustainable Behavior during the COVID-19 Lockdown: Evidence from Spanish Consumers

**DOI:** 10.3390/foods10081898

**Published:** 2021-08-16

**Authors:** Shanshan Li, Zein Kallas, Djamel Rahmani, José Maria Gil

**Affiliations:** 1 Institute for Research in Sustainability Science and Technology (IS-UPC), Polytechnic University of Catalonia, 08034 Barcelona, Spain; 2 CREDA-UPC-IRTA (Centre for Agro-food Economy and Development), 08860 Castelldefels, Spain; djamel.rahmani@upc.edu (D.R.); Chema.Gil@upc.edu (J.M.G.); 3 DEAB (Department of Agrifood Engineering and Biotechnology), Polytechnic University of Catalonia, 08860 Barcelona, Spain

**Keywords:** COVID-19 lockdown, food preferences, risk preference, risk perceptions, food purchasing behavior, food consumption behavior, sustainable behavior

## Abstract

The COVID-19 pandemic poses a threat to global food security, and it changes consumers’ food buying and consumption behavior. This research not only investigates trends in Spanish consumers’ general food shopping and consumption habits during the lockdown, but also investigates these trends from the perspective of sustainable purchasing. Specifically, total food consumption (C), food expenditure (E), and purchase of food with sustainable attributes (S) were measured. Data were collected from a semi-structured questionnaire which was distributed online among 1203 participants. The logit models showed that gender, age, employment status, and consumers’ experiences were associated with total food consumption and expenditure during the lockdown. In addition, consumers’ risk perceptions, shopping places, trust level in information sources, and risk preference were highly essential factors influencing consumers’ preferences and sustainable behavior. Consumers’ objective knowledge regarding COVID-19 was related to expenditure. Furthermore, family structure only affected expenditure, while income and place of residence influenced food consumption. Mood was associated with expenditure and the purchase of sustainable food. Household size affected purchasing behavior towards food with sustainable attributes. This research provides references for stakeholders that help them to adapt to the new COVID-19 situation.

## 1. Introduction

Novel coronavirus disease, named “COVID-19” by the World Health Organization (WHO), was initially reported in the city of Wuhan, China in December 2019 [1]. Subsequently, it began rapidly spreading around the world, resulting in a global pandemic. Spain took many preventive measures, including lockdowns, stay-at-home orders, mass quarantine, and transportation halts when COVID-19 started to spread in Spain. The Spanish government declared a state of emergency on 14 March 2020 and increased the severity of the state of alarm from 30 March to 14 April 2020, which was a strict lockdown period. People could only leave home when they were working in essential services (health, security, social, and economic wellbeing of citizens) or when they needed to buy necessary products (groceries and medicine) during the lockdown [2]. The COVID-19 pandemic situation not only affected human health, but also caused several economic and social changes. On the one hand, the rate of unemployment increased and financial strain became more severe [3], which led to an increase in depression risk, stress, and feelings of helplessness [4]. On the other hand, the COVID-19 pandemic created new working and family situations (e.g., teleworking, e-learning, homes with narrow space, and living space without direct access to sunlight), which also induced stress and depression [5].

In this context, a significant share of consumers increased their food consumption due to higher anxiety levels [6]. A previous study showed that consumers in ten European countries consumed more food as a result of the COVID-19 lockdowns across Europe and an increase in homeworking that led people to spend more time at home, influencing their consumption behavior and food choices [7]. Another study reported that almost half of the respondents stated that they increased food consumption during the lockdown in Italy, with twenty percent of them gaining weight [6]. On the contrary, compared to the period before the COVID-19 outbreak, Polish youth had a better dietary intake during the outbreak, as the pandemic changed the determinants of food choices, reinforcing the importance of health and weight control [8]. The Italian lockdown allowed consumers to make positive habits towards food consumption [9]. In addition, the COVID-19 outbreak led Spanish consumers to adopt a healthier eating habit/behavior, as evidenced by a higher level of adherence to the Mediterranean diet (MedDiet) [10].

Additionally, the COVID-19 lockdown also changed consumers’ shopping behavior. Individuals focused on buying food items as a behavioral reaction to feelings of stress and uncertainty [11]. Negative feelings (e.g., fear, stress, and uncertainty) could cause a panic buying situation [12,13]. Panic buying behavior exacerbates stock-out situations and often leads to a price increase in food products [12]. Spanish consumers were shown to be stockpiling non-perishable food and other supplies during the COVID-19 lockdown [14]. Some people stockpiled food items and bought more on each trip to minimize store visits, aiming to reduce the risk of infection [15]. According to previous research, 64% of consumers experienced product shortages at stores from which they were attempting to purchase, and 50% of consumers stocked up on products to avoid deficiencies in the future during the COVID-19 outbreak in India [16]. Additionally, consumers’ food spending increased dramatically during the COVID-19 outbreak [17,18], and another report indicated that grocery spending increased in Spain due to COVID-19 [19]. Furthermore, the COVID-19 pandemic enabled people to turn to purchasing food products online in an attempt to limit their perceived risk of exposure to infection [20]. Moreover, a previous study indicated that consumers turned to purchasing organic food or buying food products directly from farmers [21].

In addition, consumers’ shifts to more sustainable behavior can dramatically reduce their carbon impact [22], which contributes to the achievement of sustainable development in Spain. There is considerable literature that has explored consumers’ attitudes, purchasing, and consumption behavior towards food products with sustainable attributes (e.g., organic food, animal welfare food, fair-trade food, environmentally friendly food, and local food) before the COVID-19 lockdown [23,24,25,26]. However, little research attempted to measure them during the lockdown, and it is of great importance and necessity to conduct such a study that ensures the availability of sustainable food in the market during the pandemic. To date, few studies have explored how COVID-19 affected Spanish consumers’ purchasing or consumption behavior [2,27], and these studies focused on the evolution of people’s information searches or only on food consumption/dietary behavior. Evidence on trends in expenditure and purchases of food with sustainable attributes during the Spanish lockdown and their related determinants is insufficient. This research includes more comprehensive potential impact factors and, to our knowledge, is the first study that not only investigates trends in Spanish consumers’ general food buying and consumption behavior during the lockdown, but also investigates these trends from the perspective of sustainable purchasing. In this context, the main objective of this study is to analyze trends in consumers’ food preferences and sustainable behavior during the COVID-19 lockdown. To reach the main objective, three secondary objectives were proposed as intermediate steps. Firstly, this study aimed to measure changes in consumers’ total food consumption, which reflects a preference for consumer behavior during the lockdown, and to identify its determinant factors. Secondly, this study aimed to explore how consumers’ food expenditure (representing a behavioral preference) changes and to identify its impact factors. Thirdly, this study aimed to examine trends in shopping behaviors toward food attributes with sustainable behaviors.

## 2. Literature Review

A large number of studies have been conducted on the determinants of consumers’ food purchases and consumption. In summary, these can be divided into four dimensions: (1) food-related characteristics (e.g., appearance, packaging, label, and price); (2) individual socio-demographic variables (e.g., age, gender, household size, family structure, and income); (3) psychological factors (e.g., mood); and (4) cognitive factors (e.g., attitude or preference, beliefs, trust, perception, and knowledge) [28]. In this research, we focused on consumers’ socio-demographic characteristics, as well as psychological and cognitive factors.

### 2.1. Socio-Demographic Factors

Numerous studies have shown that socio-demographic characteristics have a significant impact on food purchasing and consumption behavior [23,29,30]. For example, many studies have suggested that females purchase sustainable food more frequently [31,32]. This may be because, on the one hand, women are more health-conscious, and they consider sustainable food (e.g., organic food) to be healthier than conventional food [29]. On the other hand, women are often responsible for household food purchases and are therefore more aware of sustainable food [32]. However, another study showed that although women have positive attitudes towards organic vegetables, there is no significant impact on the actual consumption of organic vegetables [33]. These different findings may be related to the attitude-behavior gap.

In some previous empirical studies carried out in Europe, income is identified as a factor influencing the purchase of organic food, for example, consumers with higher incomes are more likely to purchase higher levels of organic food [23,34]. Conversely, a study conducted in the United States did not find an association between income and organic food purchasing behavior [35]. These different outcomes may be related to regional differences. Moreover, another study suggested that income has no influence on the regularity of organic food consumption, but it affects individual spending on organic food [30].

With regard to age, previous research has shown that young consumers (18–32 years old) frequently buy organic food [32]. However, another study revealed that older consumers (over 55 years) purchase sustainable food more often [31]. Additionally, older people spend less on food, which may be related to the fact that calorie requirements decrease with age; therefore, older consumers buy a lower amount of food [36]. In addition, previous literature suggested that income and age are indicators of food spending behavior [37]. In terms of family structure, previous research has demonstrated that consumers who have children tend to buy sustainable products [38]. Another study also indicated that the presence of children in the household is positively associated with the likelihood of consuming organic food [33] and food expenditure [39]. Moreover, another study also showed that household income, size, and composition (with children) positively affect food expenditures [39]. Therefore, according to these previous findings and the ongoing global novel coronavirus pandemic, this research explored the influence of consumers’ profiles on purchasing and consumption behavior during the lockdown.

### 2.2. Psychological Factors (Mood)

Mood is one motive which may drive consumers’ food choices [40,41]. Some studies have explored the relationship between mood and food, and they have found that negative moods positively influence food intake [42,43]. In addition, an early study revealed that people are more likely to consume healthy foods in positive moods and are more prone to eat unhealthy foods (e.g., snacks high in sugar and salt) in negative moods [44]. This may be because foods high in sugar or fat can reduce the effects of negative emotions through the neurotransmission of dopamine, making people happier [45].

In contrast, Mehrabian and Riccioni pointed out that a positive mood is related to high appetite levels [46]. Another review study also indicated that positive mood is a neglected trigger for eating more food due to the close correlation between socialization and food consumption [47,48]. In particular, in terms of positive emotions, research has shown that consumers may eat more pleasantly and extend time duration of the meal, and therefore consume more food, when eating with familiar and friendly people [48].

Moreover, changes in food consumption behavior due to fluctuations in emotional states may be triggered by situations or events outside of a person’s daily routine, such as adapting to certain environments or motivating themselves [49]. Therefore, given the current global pandemic, consumers’ emotional states fluctuate and may change consumption behavior; therefore, we explored whether emotional states have an impact on consumers’ food preferences and sustainable behavior defined in this research.

### 2.3. Cognitive Factors

#### 2.3.1. Trust in Information Sources

Trust is regarded as an important predictor of consumers’ attitudes and food behavior [50]. Previous research has revealed that information from highly trusted sources is more likely to evoke changes in attitudes and behaviors [51]. Trust in information sources influences consumers’ attitudes and purchase intentions during a food security crisis [52]. Consumers’ level of trust and source of information during a health crisis may influence whether they adopt certain recommended food safety behaviors [53]. In addition, a previous study found a relationship between trust in information sources and risk perceptions [54]. Trust in authorities’ sources of information is vital to reduce unnecessary fear and inappropriate risk perceptions [55], while trust in the information given by the media increases risk perception [54]. Moreover, panic buying (increased purchasing) has occurred in public health emergencies since ancient times [56]. For example, the COVID-19 pandemic generated fear of scarcity among consumers, which increased risk perception and ultimately lead to impulsive and panic buying behavior [57]. Very little research has studied the relationship between trust in information sources regarding COVID-19 and buying behavior during the lockdown. Therefore, this research filled this gap by including trust in information sources regarding COVID-19 to measure food preferences and sustainable behavior.

#### 2.3.2. Risk Perceptions and Risk Preference

In social science research, “risk” is defined as the likelihood of physical, social, or financial harm/loss due to a hazard within a specified time frame [58]. A “hazard” is a situation, event, or substance that may be harmful to people, nature, or man-made facilities, whereas a risk is not; it is an inference about the impact of a hazard on people (or nature or assets) [59].

Risk perception plays an essential role in consumers’ purchase intentions and behavior [60], and it is defined as people’s subjective judgments about the frequency and severity of a particular risk [61]. Typically, risk perception is measured by asking participants about specific risk scenarios [61]. Several studies have shown that risk perception is an indicator of food consumption. For instance, increased risk perception of fish-eating negatively affects total consumer fish consumption [62].

Risk preference includes three types: risk-loving, risk-neutral, and risk-averse. For risk-averse consumers, sustainable attributes of food (e.g., food being organic) are risky and uncertain. Therefore, they prefer to buy and eat conventional products rather than purchase sustainable food [63]. Risk perception and risk preference (elicited through the lottery game) are key determinants of the acceptance of risky foods [64].

#### 2.3.3. Knowledge

Knowledge is a crucial strategy for consumers to make purchase decisions [65]. There are three types of knowledge: subjective knowledge (self-perceived knowledge), objective knowledge (the content of knowledge), and usage experience [66]. Several studies showed that higher levels of COVID-19 knowledge are related to changes in dietary habits and depression [67]. Lower knowledge of COVID-19 is associated with COVID-19-related behavioral changes, such as purchasing more goods and stockpiling [68,69]. In addition, knowledge may potentially affect personal perceptions and purchasing decisions, especially when health issues like COVID-19 arise [70].

Based on existing literature, we introduced these variables as potential predictors influencing consumers’ changes in food preferences and sustainable behavior, as defined in this research.

## 3. Materials and Methods

### 3.1. Data Collection and Questionnaire Design

A semi-structured questionnaire in an online survey (Qualtrics consumers’ panels) among 1203 participants was conducted during the lockdown situation in Spain in May 2020. The questionnaire for this study was divided into seven sections: (1) changes in consumers’ behavior during the lockdown, including food consumption, food expenditure, purchase of food with sustainable attributes, and shopping places; (2) consumers’ trust level in information sources; (3) risk preference; (4) risk perceptions; (5) knowledge level; (6) mental status (mood states and concerns regarding COVID-19); and (7) socio-demographic variables. The questionnaire was reviewed and validated by a group of experts from different universities and countries. The Cronbach’s alpha coefficients of the scales were tested, and all coefficients were above 0.68, indicating acceptable internal consistency. Factor analysis also confirmed the validity of the constructs. In order to have a representative sample, quota sampling stratified by age and gender was used. Consumers who were fully or partially responsible for purchasing food (over the age of 18) were recruited to participate in the present study. The questionnaire was available in Spanish. On average, each respondent spent 25 minutes filling out the questionnaire. Respondents participated in our survey voluntarily, and we explained to them the purpose of the study and that their information would not be disclosed. The questionnaire was approved by the Ethics Committee of the Centre for Agro-food Economy and Development (CREDA) and was carried out in accordance with the ethical norms of social science research.

### 3.2. Independent Variables Included in this Research

#### 3.2.1. Risk Preference

Risk preference was a highly important factor in consumers’ behavioral intention [71]. The MPL (Multiple Price List) has been widely used in psychology and economics research because of its easy and effective procedure, which was based on expected utility theory (EUT) [72,73]. Therefore, MPL was employed to measure consumers’ risk preference in this research. In this MPL experiment, respondents were asked to choose between lottery A and lottery B twenty times. In the first task, they had a 100% chance of receiving €200 under lottery A; under lottery B they had a 50% chance of receiving €200 and a 50% chance of receiving nothing. By that analogy, 20 tasks, until lottery A with a 100% chance of receiving €10, and lottery B with the same, were conducted to measure consumers’ risk preference. The payoff of lottery A decreased in turn, while the payoff of lottery B remained unchanged (€100). Lottery A is the “safe” choice whose payoff is more than the potential payoff in the “risky” lottery B among the top ten choices. In the 11th task, the payoff of lottery A is the same as that of lottery B. Starting from the 12th task, lottery A has less payoff than lottery B.

The number of “safe choices” (choosing lottery A) has often been used to describe risk preference [64]. In our research, the number of risk-loving individuals’ “safe choices” should be less than or equal to 9, while the number of risk-neutral people’s “safe choices” should be equal to 10, and the number of risk-averse people’s “safe choices” should be more than or equal to 11.

#### 3.2.2. Risk Perceptions

As concluded in the literature review, risk perception played an essential role in consumers’ purchase intentions and behavior [60]. In this research, risk perception consisted of three aspects: risk of COVID-19, food security risk, and financial risk perceptions.

As for risk perception of COVID-19, previous studies indicated that risk perception was designated as a mix of perceived vulnerability (how likely a person thinks he/she will contract the disease) and perceived severity (how serious people think contracting the disease will be for him/her) [74], which was applied in a recent study to measure perceived risk regarding COVID-19 [75]. According to the previous research measuring SARS-related risk perceptions during the 2003 SARS outbreak [76] and another study during the 2009 H1N1 pandemic [74], we measured consumers’ risk perception of COVID-19 by two items: (1) perceived risk of vulnerability, employing a 5-point Likert scale that ranges from 1 (very unlikely) to 5 (very likely) (How likely do you think you are to contract coronavirus in the next six months?); and (2) perceived risk of severity, using an 11-point Likert scale from 0 (not serious at all) to 10 (very serious) (How serious do you think your health will be if you contract the coronavirus in the next six months?). If consumers perceived a higher severity or a higher likelihood of contracting the virus (get a higher score on the 11-point or 5-point Likert scale), they had a higher risk perception of COVID-19. The 11-point Likert scale provided respondents with a wider range of options and yielded better predictive analysis. Additionally, previous research indicated that the 11-point Likert scale from 0 to 10 was popular due to its high composite reliability [77].

According to the recognized definition, food security was defined as “access to adequate food for all people at all times to have an active and healthy life” [78]. In this research, consumers’ perceived food security risk was elicited using a 7-point Likert scale that ranges from 1 (very unlikely) to 7 (very likely), and they needed to answer how likely they thought it was that food shortages and food prices would rise in the next six months (How likely do you think it is that the following scenarios will occur in the next 6 months?—food shortages; food prices will go up). Regarding financial risk, a 5-point Likert scale ranging from 1 (not at all) to 5 (a great deal) (How threatened do you feel about your current financial situation?) was used.

#### 3.2.3. Mood States, Experiences, Concerns, and Shopping Places

As introduced in the literature review, negative and positive moods influenced food choices [79]. COVID-19 put consumers under great stress and caused them to exhibit different moods, which may have influenced their purchasing and consumption behavior during the pandemic. Therefore, respondents were asked about their mood status (including positive moods and negative moods) via a 5-point Likert scale ranging from 0 (none of this feeling) to 4 (a great deal of this feeling) (Considering the COVID-19 situation where you currently live, do you feel…?—irritated; confident; angry; reassured; annoyed; and aggravated). Anger as a negative mood was measured using four items that had been validated in previous studies: irritated, angry, annoyed, and aggravated [80]. Cronbach’s alpha was α = 0.91 in this research. The positive emotions included feeling reassured and confident, which were selected from previous research [81]. Cronbach’s alpha was α = 0.80.

In addition, the COVID-19 pandemic brought stress and uncertainty for people, which could result in panic buying, thus threatening global food security. For people who experienced food shortages or higher food prices during the COVID-19 outbreak, their purchasing behavior may have changed [82]. As a consequence, in this study, we measured food security experiences (food shortages, higher food prices, and neither) (During the outbreak, did you experience the following scenarios?—food shortages; higher food prices; and neither of them). In addition, we measured COVID-19 experiences, similar to a recent study [83], by asking respondents if they contracted COVID-19 or not (Have you contracted the COVID-19 virus?) (1 = Yes, I tested positive for the COVID-19 virus; 2 = No. I had the symptoms, but the test result came back negative; 3 = No. I did not have the symptoms, so I did not opt for a test; and 4 = I do not know. I had the symptoms but did not have access to a test) and asking if they knew someone who had been diagnosed or died due to COVID-19 (Do you know someone who has been diagnosed or died due to the COVID-19 virus?—members of my family; friends; neighbors; friends of my friends; colleagues; and no, I don’t know any person) and examined whether experiences played an important role in consumers’ behavior during the lockdown.

Additionally, previous work indicated that consumers’ concerns were related to buying behavior [84]. Concerns regarding COVID-19 were wide-ranging, encompassing both health and financial issues [85]. Hence, we adopted a 7-point Likert scale ranging from 1 (not concerned at all) to 7 (extremely concerned) to evaluate consumers’ health concerns about COVID-19 and ultimately to explore its impact on consumers’ behavior during the lockdown (Please indicate your level of health concern about COVID-19). In addition, a previous study showed that there was a significant increase in food shopping online, with 45% of consumers in ten European countries making more online purchases during the lockdown [7]. Another study conducted in South Korea indicated that during the 2015 MERS outbreak in South Korea, consumers decreased their spending on food at department stores and outside the home, while they increased their spending on food purchased online, suggesting that changes in shopping location influenced changes in consumers’ food expenditure [86]. Moreover, a shopping place, such as a large store, can stimulate consumers’ emotions, which can further influence purchase decisions [87]. Therefore, change in shopping place as a potential indicator was included in this research. Respondents were asked to answer two questions to assess the shopping place variable before and during the lockdown (Where do you usually buy food products? (Before restrictions due to COVID-19) and Where do you usually buy food products? (During the lockdown)) (1 = hypermarkets, supermarkets; 2 = specialized food stores; 3 = malls; 4 = farmer’s market/open markets; 5 = retailers’ websites; 6 = organic food stores; and 7 = others).

#### 3.2.4. Trust in Information Sources and Knowledge

Consumers look for health information from a wide cluster of sources and channels [88]. Trust in health organizations and government health agencies has been identified as an important correlate of health-related decision-making and behavior [89]. In public health emergencies (e.g., a flu flare-up), people with high trust in government health agencies react more rapidly and are more likely to comply with the health recommendations given by the agencies [90]. As mentioned in the literature review, trust in information sources influenced consumers’ attitudes and purchase intentions during a food security crisis [52]. In this context, consumers’ trust in information sources was elicited by using a 5-point Likert scale ranging from 1 (not trustworthy at all) to 5 (extremely trustworthy) (Consider the following sources of information regarding COVID-19. How trustworthy do you feel these sources are?—government; social media such as Twitter, Facebook; health professionals such as doctors; family, friends, and colleagues; scientists; and news such as papers, TV, and radio).

In addition, we assessed consumers’ levels of subjective and objective knowledge regarding COVID-19 to determine if they have an impact on their shopping and consumption behavior. To be specific, a 7-point Likert scale ranging from 1 (not knowledgeable at all) to 7 (very knowledgeable) was employed to measure respondents’ subjective knowledge (How well do you think you know COVID-19), with the results expressed as a percentage, i.e., from 0 (not knowledgeable at all) to 100 (very knowledgeable). In addition, the level of objective knowledge was displayed as the percentage of correct answers, and respondents were asked to judge whether symptoms of COVID-19 were correct or incorrect; symptoms presented included existing and non-existing symptoms (The following are 17 symptoms of COVID-19. Please judge whether they are true or false).

### 3.3. Measuring Consumers’ Food Preferences and Sustainable Behavior

In this research, three dependent variables, including changes in total food consumption (C), food expenditure (E), and purchasing behavior towards food with sustainable attributes (S), were measured to determine trends in food preferences and sustainable behavior during the lockdown. Changes in food consumption and expenditure were measures of food behavioral preferences during the lockdown. Respondents were asked to answer a question (How has COVID-19 impacted your total consumption of food), reflecting consumers’ consumption behavior during the lockdown. Individual scores ranged from “−3” (greatly decreased) to “+3” (greatly increased) regarding total food consumption (C). In addition, respondents were asked to respond to a question (How has COVID-19 impacted your food shopping behavior?—spending money on food purchases), with scores ranging from “−3” (greatly decreased) to “+3” (greatly increased) regarding food expenditure to measure consumers’ purchasing behavior (E). Consumers’ sustainable purchasing behavior (S) was assessed by their purchases of sustainable food (organic, local, animal welfare, and fair-trade food), with scores ranging from “−3” (greatly decreased) to “+3” (greatly increased) (During the COVID-19 lockdown, how did your purchases of the following foods change?—organic; local; animal welfare; and fair-trade). Cronbach’s alpha was α = 0.68.

**Figure 1:**
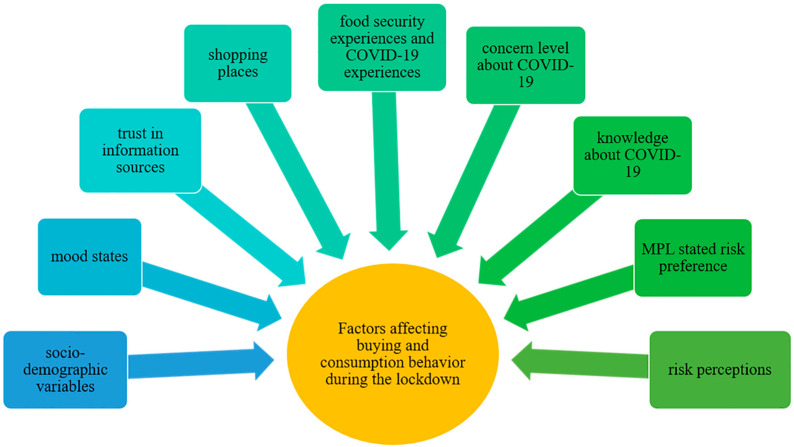
The framework of factors affecting consumers’ food buying and consumption behavior during the lockdown.

Figure 1 illustrates the framework of this study. The independent variables included in this study are the factors mentioned earlier that may be associated with consumers’ food shopping and consumption behavior. Table 1 presents the details of the sample profile. The Kolmogorov–Smirnov test was used to measure the normality of the variables, and the mean and standard deviation (SD) were computed.

As can be seen, among the 1203 respondents, 51.0% were females, and 57.0% stated that they were healthy. 56.1% of respondents (before the lockdown) and 53.6% (during the lockdown) had a monthly household income of 1000–3000 euros, and the majority were aged 40–59 years (36.9%). In addition, 36.3% of samples had a household size of 2 people, and 61.2% of households had no children aged 0–12 or adults aged over 70 years. 71.8% of participants lived in urban places, and 24.4% had a full-time job (without variation). According to the gender and age distribution, the sample reflected the population of Spain.

### 3.4. Data Analysis

In this study, consumer behavior change (the dependent variable) was a dichotomous variable with two categories: increase and no increase. The logit regression has often been used to analyze discrete dependent variables; therefore, the binary logistic regression model was deemed appropriate for this study. SPSS version 24.0 (IBM, Chicago, IL, USA) software was used. A descriptive analysis was also employed.

## 4. Results and Discussion

### 4.1. Results of the Independent Variables Included in the Model

Table 2 presents the results of the independent variables included in the model. Results revealed that Spanish consumers’ subjective and objective knowledge level regarding COVID-19 was above average (77.26% > 50.00% and 67.44% > 50.00%). This may be due to the fact that the Spanish government, health experts, and the media have conveyed a considerable amount of information about COVID-19 to society. The results also showed that 65.7% of respondents were risk-averse, 13.6% were risk-neutral, and 20.7% were risk-loving. This result is in line with previous studies showing that the majority of respondents were risk-averse [91], and only a small proportion of participants were risk-loving [92]. In addition, with regard to food security experiences, 29.2% of participants stated that they experienced a food shortage during the lockdown, and 60.7% of them experienced rising food prices. As for COVID-19 experiences, the results showed that 71.7% of respondents stated that they did not have symptoms, so did not opt for a test. Only 1.5% of respondents tested positive for the COVID-19 virus. 21.7% of consumers did not know due to no access to a test. 37.2% of respondents did not know anyone who had been diagnosed or died due to the COVID-19 virus.

Participants’ concern level about COVID-19 was above average (4.77 > 3.5 points on a 7-point scale), which is consistent with research showing that levels of concern about COVID-19 are relatively high in Spain [93]. The probability of consumers perceiving food shortages in the next six months was below average (2.34 < 3.5 points on a 7-point scale). With respect to the probability of facing higher food prices in the next six months, this was perceived to be above average (5.01 > 3.5 points on a 7-point scale). The news reported that in Spain, fruit and vegetables have become between 25% and 30% more expensive due to the increase in transport costs during the COVID-19 pandemic [94], which has increased consumers’ perceived food price (food security) risk. In addition, this was supported by the result of “experiences” in this research (as shown earlier), which showed that 60.7% of consumers experienced a higher food price during the lockdown, increasing their food security risk perceptions. Consumers’ experiences of food insecurity will increase their risk perception because direct exposure to risk events usually enhances consumers’ memories and imaginations of hazards [95].

As for the severity of the perceived risk, this was above average (6.04 > 5.5 points on an 11-point scale). Regarding the probability of contracting COVID-19 in the next six months, the results indicated that consumers assessed their risk of being infected as high (2.65 > 2.5 points on a 5-point Likert scale). In both cases, the scores were slightly above the average, indicating a slightly higher perceived risk regarding COVID-19. These outcomes converge with the findings that Spain was the second country with the highest risk perception of COVID-19 among ten countries across Europe, America, and Asia [93]. Consumers’ trust level in information sources from the highest to lowest was health professionals, scientists, family (friends and colleagues), social media, government, and news. This is in line with a study which concluded that consumers stated information from experts or scientists was the most reliable [2].

### 4.2. Results of Consumers’ Food Preferences and Sustainable Behavior

According to Table 3, the majority of the respondents stated that they did not increase total food consumption (63.8%) or purchase more food with sustainable attributes (55.2%) when compared to the situation before the lockdown. However, the majority of respondents (52.6%) stated that they increased food expenditure during the lockdown.

#### 4.2.1. Changes in Total Food Consumption (C) during the Lockdown

As reported in Table 4, the percentage of the model’s correct classification was 75.2%, and the Hosmer–Lemeshow’s goodness of fit was equal to 0.353, leading us to accept the null hypothesis that there was no significant difference between the observed and model-predicted values [96]. The OR of gender was equal to 1.394, meaning that females were 1.394 times more likely to increase food consumption than males during the lockdown. One possible reason was that many food-away-from-home establishments were closed because of the shutdown restrictions during COVID-19 in Spain, such that an increasing number of working women had to cook at home, where they tended to consume more food. Another reason may be that women were more prone to depression, stress, and anxiety than men, resulting in more emotional eating [97].

In addition, people aged 40–59 years and more than 60 years old were less likely to increase total food consumption than those aged 18–39 years when compared to the situation before the lockdown. This is in line with a study which showed that older people consumed less than younger people during the COVID-19 lockdown [98]. The results also demonstrated a positive and significant association between income and total food consumption. This indicated that households whose monthly income before the lockdown was more than 3000 euros were 2.963 times more likely to increase total food consumption than those less than 999 euros. Not surprisingly, more income in a household denoted a stronger purchasing power to provide food for their family members, such that they were more likely to increase total food consumption during the COVID-19 lockdown. People whose current employment status was ERTE (partial or total), on sick leave, unemployed, or unable to work were less likely to increase their food consumption during the lockdown. It was expected that these people’s jobs were suspended or they were unable to work, such that their sources of income were cut off by COVID-19, and they were less likely to increase their consumption level. However, there was little change in income (no income) for students before and during the lockdown. Results also indicated that people who live in rural places were less likely to consume more food than those living in urban places. This may be related to several reasons. Firstly, population flow is more frequent in urban areas than that in rural places, resulting in a higher risk of contracting COVID-19 for consumers who live in urban areas. Consequently, people living in urban places may feel worried, anxious, or negative about themselves; thus, they tended to display emotional eating behavior to avoid these negative feelings by turning their attention to food during the lockdown [99]. Secondly, consumers living in urban areas usually have a higher income than those living in rural places; that is, they have a stronger purchasing power and consumption power.

As for consumers’ stated risk preference, the results showed that risk-averse people were less likely to increase their total food consumption than risk-loving persons. A previous study indicated that risk-averse respondents may seek out more insurance after a disaster [100]; thus, risk-averse people may focus on health insurance or save money to make themselves feel more secure and use it when there is a health threat in the future. Respondents who did not experience food shortages or higher food prices or did not know someone who had been diagnosed or died due to COVID-19 were less likely to consume more food than those who experienced these situations. This could be explained by the fact that subjects who experienced food shortages or higher food prices or knew someone who had been diagnosed or died due to COVID-19 were more likely to be anxious [101]; thus, they were prone to emotional eating (over-eating). Regarding shopping places, people who went to specialized food stores and farmers’ markets to purchase food before the lockdown were less likely to consume more food than those who went to supermarkets. This may be because specialized food stores and farmers’ markets only sell food, while supermarkets have a wider variety of not only food products but also other necessities, such as toilet paper, shampoo, and pet supplies. Therefore, in order to reduce the number of visits to stores and reduce the risk of infection, consumers who used to buy food from specialized food stores and farmers’ markets may have preferred to buy food from supermarkets during the lockdown, such that those who went to supermarkets consumed more food.

Results also showed that consumers were less likely to increase their food consumption when they perceived a higher trust level in health professionals (e.g., doctors) during the lockdown. Trust in reliable scientific information contributes to reducing unnecessary scares and inappropriate risk perceptions [55]. Hence, consumers who trust health professionals could reduce their risk perception and were less likely to panic buy and consume food. Regarding risk perception of COVID-19, this category demonstrated that consumers who perceived a higher risk of COVID-19 were more likely to increase their total food consumption than those who perceived a lower risk during the lockdown. This may be because if consumers thought the situation was serious, they were worried about themselves and tended to display emotional eating behavior. As for food security risk perception, this category revealed that consumers who perceived a higher risk of food shortages in the next six months were more likely to increase total food consumption than those perceiving the lowest food security risk. It was not surprising that people with a higher food security risk perception tended to stockpile food products to reduce the food security risk; thus, they turned to increase food consumption.

#### 4.2.2. Changes in the Total Food Expenditure (E) during the Lockdown

In Table 5, the percentage of correct classification was 70.3%, and the value of Hosmer–Lemeshow’s goodness of fit was 0.311, indicating that the model presented an acceptable goodness of fit. The results demonstrated that females were less likely to spend more on food than males during the lockdown. The data from the National Statistics Institute in Spain showed that the unemployment rates of females and males in the first quarter of 2020 in Spain were 16.24% and 12.79%, respectively. In the second quarter, they stood at 16.72% (females) and 14.13% (males) [102], indicating that females had a higher likelihood of being unemployed than males during the lockdown. Hence, females were more cautious about their income and less likely to increase food expenditure. Another potential reason was that females were the main meal preparers and “food gatekeepers” in the household [103]. As a result, they were more familiar with the characteristics (e.g., the price and the quality) of food products and always knew what food to buy, such that females were less likely to increase food expenditure. Conversely, males were not usual food buyers and not familiar with food products; therefore, males may have increased their expenditure on food.

People aged 40–59 years and more than 60 years old were less likely to increase expenditure than those aged 18–39 years when compared with the situation before the lockdown. The elderly were at a high risk of death due to COVID-19, which may have increased their worry and further affected their appetite [104]. Therefore, their cost was not likely to increase compared to younger people during the COVID-19 lockdown. Results also indicated that respondents whose employment status was sick leave and unable to work were less likely to spend more on food during the lockdown, which may be related to the interruption of their income. In addition, households with children aged 7–12 years were 2.218 times more likely to increase food expenditure than those without children. It was expected that primary schools were closed during the lockdown, such that children aged 7–12 years had to stay at home, resulting in more expenditure. Participants who experienced food shortages during the COVID-19 lockdown were 1.688 times more likely to increase their food expenditure than those who did not face food shortages. If consumers had experienced food shortages, they were likely to perceive that future food supplies may also be limited. Therefore, they spent more and stockpiled more food to reduce food security risks. In addition, consumers who tested positive or knew someone who had been diagnosed or died due to COVID-19 were more likely to increase food expenditure. This may be attributed to the fact that these people perceived a higher risk of contracting COVID-19. They therefore tended to buy more food per visit and reduce the number of shopping trips, thus reducing the risk of infection and consequently spending more on food. As for shopping places, consumers who bought food on retailers’ websites during the lockdown were 4.574 times more likely to spend more on food than those who bought food in supermarkets. This is consistent with a study which found a significant increase in online shopping due to COVID-19 [7]. It was expected that consumers tended to shop online rather than in supermarkets to minimize store visits, aiming to reduce the risk of infection.

In addition, our results demonstrated that consumers with a positive mood (reassured) were more likely to increase food expenditure, while those with a negative mood (angry) were less likely. This outcome is supported by Mehrabian and Riccioni, who concluded that a positive mood was associated with high appetite levels [46]. Therefore, people with a positive mood during the lockdown tended to purchase more food and increase food expenditure, while a negative mood decreased consumers’ appetite; thus, they were less likely to increase food expenditure. With regard to risk preference, the results implied that risk-neutral and risk-averse people were less likely to increase their food expenditure than those who were risk-loving during the lockdown. This may be related to risk-averse people’s aversion to uncertainty, i.e., risk-averse consumers prefer certainty to uncertainty more than risk-loving ones. Due to the COVID-19 outbreak, they may tend to reduce food expenditure and save more money to prevent insufficient money when uncontrollable situations arise in the future. The findings also revealed that consumers were less likely to spend more on food when they perceived greater trust in government and news information regarding COVID-19 during the lockdown. This is supported by a study which demonstrated that higher trust in the national government had positive effects, such as reducing the likelihood of respondents’ fears and worry of food shortages [105]. Consequently, these consumers perceived a lower food security risk and were less likely to stock up on food and increase food expenditure.

As for consumers’ risk perceptions, the results indicated that the higher the COVID-19 risk and food security risk the consumers perceived, the more expenditure was seen. This is in line with a study which showed that consumers tend to purchase more stock goods when they perceive a higher risk, and this also indicates that a high risk perception during the COVID-19 pandemic will cause the intention to buy goods, leading to a higher probability of increasing food expenditure [106]. Another study also demonstrated that risk perception of the COVID-19 pandemic has positively affected consumers’ behavior regarding the tendency to maintain food stocks [107]. The results also showed that consumers would not increase food expenditure when they perceived a higher financial risk, which highlighted previous research showing that risk perception negatively affected attitude and purchasing behavior [108]. This was expected, because when consumers feel threatened about their current financial situation, that is, perceiving a higher financial risk, they are more cautious about spending money. Additionally, consumers with a higher objective knowledge level regarding COVID-19 were found to have a higher likelihood of increasing food expenditure. It was expected that the more knowledge consumers had, the more severity about COVID-19 they perceived, such that they were more likely to increase expenditure to stock up on food.

#### 4.2.3. Changes in Purchasing Food with Sustainable Attributes (S) during the Lockdown

As shown in Table 6, the fit was acceptable as indicated by Hosmer–Lemeshow’s goodness of fit measures and the percentage of correct classification. The result showed that households with 5 members were 2.551 times more likely to purchase more food with sustainable attributes than those with 1 member when compared with the situation before the lockdown. This is supported by a study which indicated that consumers living in larger households were more likely to purchase organic food products [23].

In addition, risk-averse consumers were less likely to increase their purchases of food with sustainable attributes during the lockdown. This outcome converges with the finding that risk-averse respondents avoided buying more sustainable food during the lockdown in China [109]. It may relate to the uncertainty consumers feel when uncertain about food with sustainable attributes (e.g., whether organic certification can be trusted); they may therefore prefer the certainty of conventional products to the uncertainty that may come from sustainable ones [63]. The results also indicated that people who used to purchase food from specialized food stores (before the lockdown) were less likely to buy more food with sustainable attributes than those who usually went to supermarkets. Similar to the previous explanation, one possible reason was that specialized food stores only have food, while supermarkets have a more complete variety (e.g., food, alcohol, toilet paper, and pet supplies). As a consequence, consumers who used to purchase food from specialized food stores may be inclined to buy food (including food with sustainable attributes) and other necessities from the supermarkets during the lockdown to minimize trips to the store and reduce the risk of infection. Additionally, consumers with a positive mood (reassured) were more likely to purchase more food with sustainable attributes while those with a negative mood (angry) were less likely. One possible explanation was that positive emotions make consumers perceive sustainable food (e.g., organic food) as more attractive, and they are eager to purchase and consume healthy food [110].

According to the results, consumers with a higher trust level in government were more likely to increase their purchasing of food with sustainable attributes. This is supported by a study indicating that in public health emergencies, people who have high trust in government health agencies were more likely to follow health recommendations (including food choice recommendations) made by the government [90], and they regard sustainable food (e.g., organic food) as healthier food. Thus, they are more likely to purchase more food with sustainable attributes. The results also implied that consumers with higher risk perceptions of COVID-19 and food security were more likely to buy more food with sustainable attributes. Similarly, consumers in Spain perceived these products were healthier than conventional ones [111], which contributes to improving their immunity and reducing the risk of infection. The results also demonstrated that respondents who perceived a higher financial risk were less likely to purchase more food products with sustainable attributes when compared with the situation before the lockdown. Not surprisingly, food products with sustainable attributes were more expensive than conventional food [112]. Consumers tended to buy less sustainable food (expensive) when they perceived a higher financial risk, and they would spend money more carefully during the COVID-19 pandemic. The results of food security risk perception and financial risk perception are similar to the previous research conducted in China [109], but we did not find the effects of gender and age on the purchases of food with sustainable attributes in this research.

### 4.3. Overall Discussion

Overall, the majority of respondents stated that they did not increase food consumption (63.8%) or purchase more food with sustainable attributes (55.2%) during the lockdown. This is supported by a recent study which showed that 74% of respondents in Spain did not increase their food intake [113]. However, the majority of them (52.6%) stated that they increased food expenditure during the lockdown. This may be due to the fact that most Spanish participants reduced their food purchase frequency, which led to increased expenditure for each food purchase occasion [113].

Our results showed that females tended to consume more food but with less expenditure on food than males during the lockdown. Females were more likely to be depressed, stressed, and anxious, which can lead to emotional eating [97,114]. As previously explained, on the one hand, women were more likely to be unemployed during the COVID-19 pandemic compared to men, causing them to experience financial pressure [102]. On the other hand, women were the main food buyers and gatekeepers in the household [32,103]; therefore, they were more aware of food and did not engage in more spending. The literature review concluded that women were more likely to buy foods with sustainable attributes [31,32] because they were more health conscious and perceived sustainable foods as healthier [29]. However, this research did not identify a relationship between gender and purchase of sustainable food during the lockdown in Spain. 

In this research, family structure only affected food expenditure, which is in line with an early study that showed households with children increased their food expenditure during the lockdown [39]. Nevertheless, we did not find that this factor influenced the other eating and purchasing behaviors defined in this study. From the literature review, household size positively influenced food expenditure [39], but our result indicated that household size was only a statistically significant factor affecting purchasing behavior towards sustainable attributes. Our results indicated that age was an indicator related to total food consumption and expenditure when compared to the situation before the lockdown, i.e., older people were less likely to increase food intake and expenditure. This can be explained by the fact that older people need fewer calories than younger people; thus, they buy less food and consume less [36]. This may also be related to the high risk of death in older adults due to COVID-19, which may increase their worry and fear, further affecting their appetite [104].

Mood was found to be associated with expenditure and purchasing food with sustainable attributes. This may be because, on the one hand, positive emotions make consumers perceive sustainable food (e.g., organic food) as more attractive, and they are more likely to be eager to purchase and consume healthy food [110]. On the other hand, positive emotion was correlated with high appetite levels [46], and it has been a neglected trigger for eating more food [47]. Hence, people who have positive emotions during the lockdown tended to buy more food and increase their food expenses, while negative emotions decreased consumers’ appetite, and therefore, they were less likely to increase their food spending. However, we did not find a relationship between mood and total food consumption.

In addition, consumers’ risk perceptions and trust in information sources were crucial factors in understanding consumers’ food preferences and sustainable behavior during the lockdown. To be specific, consumers increased their food consumption, food expenditure, and purchased more sustainable food when they perceived a higher risk of COVID-19 and food security. However, consumers were less likely to increase expenditure and sustainable food when they perceived higher financial risks. This was in accordance with our expectation that, as explained earlier, food with sustainable attributes was more expensive than conventional food, and these people were more careful in spending their money [112]; thus, they were less likely to increase purchases of food with sustainable attributes and food expenditure. The results of food security risk perceptions and financial risk perceptions were comparable to previous studies conducted in China [109]. In addition, consumers’ trust level in information from health professionals and scientists was higher than that from the government and news. Similar findings were found in a Chinese study, where health professionals were the most trusted source about COVID-19 [115]. This is also consistent with a previous study investigating perceived trust in general health information which showed that health professionals were identified as the most trusted sources [116]. This suggested that health professionals were the most trusted source of information, both for general health information and specific disease (e.g., COVID-19) information.

The results did not identify significant impacts of subjective knowledge, concerns, or stated health status on food preferences and sustainable behavior defined in this study. These results allow the government and stakeholders to deepen their understanding of consumers’ preferences and sustainable behaviors during the lockdown in order to develop realistic policies and strategies.

## 5. Conclusions

This study explored trends in food preferences and sustainable purchasing behavior of Spanish consumers during the COVID-19 lockdown and the factors influencing them. Our empirical results gave some insights to the government, retailers, and other stakeholders that help them to adapt to the new COVID-19 situation.

### 5.1. Practical Implications

Firstly, based on the result of the increased expenditure on the retailers’ websites, retailers should design a more visually attractive and convenient website, taking advantage of this opportunity to retain customers. Secondly, the Spanish government should make efforts to design more effective information to communicate with people and should enhance the quality and level of detail of the information that they share in such an emergency. This is because consumers reported low trust in government and news while reporting high trust in health professions and scientists, inspiring health professions and scientists to share more reliable and trustworthy information about COVID-19 and recommendations of food choices and consumption. Thirdly, households with children aged 7–12 years were more likely to increase food expenditure. As a result, retailers could carry out promotion activities (e.g., children’s related food can be given as a gift if consumers spend a certain amount of money in the store), so as to attract families with children. Finally, consumers who live with large households and those who often go to the supermarket to buy food were more likely to purchase more food with sustainable attributes, reminding retailers to focus on these people by using this argument to first place and highlight sustainable items (e.g., organic items) in hotlines on the shelves.

### 5.2. Limitations and Future Research

Despite the contributions of this study, it has some limitations. Firstly, the data are based on stated rather than revealed behavior. Self-report items may be a limitation with respect to data quality, e.g., social desirability bias and lack of memory, inspiring future research with a focus on consumers’ revealed behavior. Secondly, this research explored consumers’ behavior before and during the lockdown but did not measure changes after the lockdown. Therefore, further research could explore whether this change in consumption and purchasing behavior is long-term in this global crisis, and can also explore other consumption and purchasing behaviors. Finally, the online survey excluded those who were unfamiliar with and did not have access to the internet. Nevertheless, because of its low cost and rapidity, it still gave valid data for this study, representing the population of Spain. Future studies could try to conduct face-to-face surveys after this outbreak to obtain a more comprehensive sample.

## Figures and Tables

**Table 1:** Socio-demographic variables in this research (*n* = 1203).

Socio-Demographic Variables		Percentage (%)
Gender	Male	49.0
	Female	51.0
Age	18–39 years	28.1
	40–59 years	36.9
	More than 60 years	35.0
Monthly household income before the lockdown	<999 euros	10.5
	1000–3000 euros	56.1
	>3001 euros	22.0
	“I prefer not to answer”	11.4
Monthly household income during the lockdown	<999 euros	19.0
	1000–3000 euros	53.6
	>3001 euros	15.9
	“I prefer not to answer”	11.5
Stated health status	Unhealthy	43.0
	Healthy	57.0
Household size	1 person	10.7
	2 persons	36.3
	3 persons	27.0
	4 persons	20.3
	5 persons	4.0
	6 persons or more	1.7
Family structure	There are children aged 0–6 years	Yes (13.5), No (86.5)
	There are children aged 7–12 years	Yes (15.5), No (84.5)
	There are adults over 70 years	Yes (14.1), No (85.9)
	None of the above	Yes (61.2), No (38.8)
Place of residence	Urban place	71.8
	Suburban place	14.8
	Rural place	13.4
Employment status	Student	2.3
	Full time (without variation)	24.4
	Full time (telecommuting)	16.5
	ERTE ^a^ (partial or total)	10.8
	A homemaker	5.2
	Sick leave	2.1
	Unemployed	15.0
	Retired	21.5
	Unable to work	2.2

^a^ refers to a File of Temporary Regulation of Employment (ERTE). It consists of a temporary collective dismissal, in which the company temporarily suspends employment contracts, for reasons including the temporary stoppage of activities or insufficient income.

**Table 2:** Results of the independent variables included in the logit model.

**Variables**	**Percentage (%)**	**Scales**
Knowledge		
Subjective knowledge level	77.26	1–100%
Objective knowledge level	67.44	1–100%
Risk preference		
Risk-loving	20.7	
Risk-neutral	13.6	
Risk-averse	65.7	
Experiences		
Food security experiences		
Experienced food shortages	Yes 29.2; No 70.8	
Experienced higher food prices	Yes 60.7; No 39.3	
Experienced neither	Yes 28.4; No 71.6	
COVID-19 experiences		
Q. Have you contracted the COVID-19 virus?		
Yes. I tested positive for the COVID-19 virus.	1.5	
No, I had the symptoms, but the test result was negative.	5.1	
No. I did not have the symptoms, so I did not opt for a test.	71.7	
I don’t know. I had the symptoms but did not have access to tests.	21.7	
Q. Do you know someone who has been diagnosed or died due to the COVID-19 virus?		
Members of my family	Yes 19.0; No 81.0	
Friends	Yes 26.4; No 73.6	
Neighbors	Yes 14.3; No 85.7	
Friends of my friends	Yes 25.6; No 74.4	
Colleagues	Yes 6.6; No 93.4	
No, I don’t know any person	Yes 37.2; No 62.8	
**Variables**	**Mean (SD)**	**Scales**
Concern level about COVID-19	4.77 (1.70)	7-point Likert scale
Food security risk perception		
The probability of food shortages in the next 6 months	2.34 (1.49)	7-point Likert scale
The probability of higher food prices in the next 6 months	5.01 (1.61)	7-point Likert scale
Risk perception of COVID-19		
The severity of one’s health condition will be if they contract COVID-19	6.04 (2.40)	11-point Likert scale
The probability of contracting COVID-19	2.65 (0.95)	5-point Likert scale
Trust in information sources		
Government	2.52 (1.27)	5-point Likert scale
Social media	2.70 (1.09)	
Health professionals (e.g., doctor)	4.27 (0.82)	
Family, friends, and colleagues	2.91 (1.04)	
Scientists	4.13 (0.91)	
News (e.g., papers, TV, radio)	1.90 (0.94)	

SD: standard deviation.

**Table 3:** Behavioral changes during the lockdown.

Category	Percentage		
	Total Food Consumption	Expenditure	Sustainable Food
Increase (Y = 1)	36.0%	52.6%	37.8%
Did not increase (Y = 0)	63.8%	47.4%	55.2%
Missing	0.2%	Null	7.0%

**Table 4:** Logit model of total food consumption **(C)**.

Significant Variables	Reference Category	Beta (B)	*p*-Value	Exp (B)
Gender				
Female	Male	0.332	0.063	1.394
Age				
40–59 years old	18–39 years old	−0.622	0.003	0.537
More than 60 years old		−0.977	0.001	0.376
Monthly household income				
Income (before the lockdown) >3000 euros	<999 euros	1.086	0.021	2.963
Employment status				
ERTE (partial or total)	Student	−1.061	0.080	0.346
Sick leave		−2.142	0.017	0.117
Unemployed		−1.020	0.087	0.361
Unable to work		−1.979	0.023	0.138
Place of residence				
Living in rural place	Urban	−0.437	0.077	0.646
Risk preference				
Risk-averse	Risk-loving	−0.365	0.085	0.694
Experiences				
Did not experience food shortages or price increase	Experienced	−0.785	0.026	0.456
“I know a friend of my friends has been diagnosed or died due to COVID-19”	Do not know	0.564	0.011	1.759
Shopping places				
Specialized food stores (before the lockdown)	Supermarkets	−0.750	0.021	0.473
Farmer’s market/open markets (before the lockdown)		−1.480	0.052	0.228
Trust in information sources				
Health professionals were perceived to be a little trustworthy	Not at all	−3.078	0.042	0.046
Food security risk perception				
A little unlikely to face food shortages in the next 6 months	Very unlikely	0.643	0.003	1.903
Risk perception of COVID-19				
Somewhat serious if contracting in the next 6 months	Not at all	1.595	0.003	4.930
Very serious if contracting in the next 6 months		1.596	0.012	4.934
Percentage of correct classification Hosmer–Lemeshow’s goodness of fit		75.2%		
		0.353		

**Table 5:** Logit model of food expenditure (E).

Significant Variables	Reference Category	Beta (B)	*p*-Value	Exp (B)
Gender				
Female	Male	−0.458	0.008	0.632
Age				
40–59 years old	18–39 years old	−0.572	0.006	0.564
More than 60 years old		−0.675	0.015	0.509
Employment status				
Sick leave	Student	−1.617	0.054	0.199
Unable to work		−1.485	0.060	0.226
Family structure				
There are children aged 7–12 years in the household	No	0.797	0.079	2.218
Experiences				
Experienced food shortages during the lockdown	Did not	0.524	0.017	1.688
Did not have symptoms, so did not test	Tested positive	−1.265	0.078	0.282
Did not know anyone who has been diagnosed or died due to COVID-19	Knew someone	−0.784	0.002	0.457
Shopping places				
Buy food on retailers’ websites during the lockdown	Supermarkets	1.520	0.015	4.574
Mood				
Feel a little reassured	None of this feeling	0.794	0.004	2.213
Feel moderately reassured		0.582	0.044	1.789
Feel moderately angry		−0.859	0.017	0.424
Feel a great deal of angry		−0.722	0.095	0.486
Risk preference				
Risk-neutral	Risk-loving	−0.505	0.066	0.604
Risk-averse		−0.528	0.009	0.590
Trust in information sources				
Government information regarding COVID-19 was perceived to be a little trustworthy	Not trustworthy at all	−0.425	0.092	0.654
News information regarding COVID-19 was perceived to be very trustworthy		−1.021	0.030	0.360
Food security risk perception				
A little unlikely to face food shortages in the next 6 months	Very unlikely	0.543	0.036	1.722
Risk perception of COVID-19				
A little unlikely to contract COVID-19	Very unlikely	0.819	0.004	2.268
Financial risk perception				
Feel threatened moderately about financial situation	Not at all	−0.836	0.033	0.434
Feel threatened considerably about financial situation		−0.981	0.035	0.375
Feel threatened a great deal about financial situation		−1.502	0.009	0.223
Knowledge regarding COVID-19				
A higher level of objective knowledge		0.944	0.075	2.570
Percentage of correct classification		70.3%		
Hosmer–Lemeshow’s goodness of fit		0.311		

**Table 6:** Logit model of purchasing food with sustainable attributes **(S)**.

Significant Variables	Reference Category	Beta (B)	*p*-Value	Exp (B)
Household size				
Households with 5 members	1 member	0.936	0.066	2.551
Risk preference				
Risk-averse	Risk-loving	−0.403	0.058	0.668
Shopping places				
Specialized food stores (before the lockdown)	Supermarkets	−0.710	0.028	0.492
Mood				
Feel considerably reassured	None of this feeling	0.773	0.036	2.166
Feel moderately angry		−0.953	0.010	0.386
Trust in information sources				
Government information regarding COVID-19 was perceived to be very trustworthy	Not at all	0.481	0.095	1.618
Food security risk perception				
A little unlikely to face food shortages in the next 6 months	Very unlikely	0.369	0.082	1.446
A little likely to face food shortages in the next 6 months		1.152	0.064	3.163
Risk perception of COVID-19				
A little unlikely to contract COVID-19	Very unlikely	0.748	0.015	2.113
Financial risk perception				
Feel threatened moderately about financial situation	Not at all	−0.675	0.093	0.509
Feel threatened a great deal about financial situation		−1.125	0.051	0.325
Percentage of correct classification		73.0%		
Hosmer–Lemeshow’s goodness of fit		0.095		

## Data Availability

The data presented in this study are available on request from the corresponding author.
